# The Apemen Faces Database (ApeFD)

**DOI:** 10.1038/s41597-025-05813-z

**Published:** 2025-08-21

**Authors:** Zdzisław Lewandowski, Slawomir Wacewicz, Juan Olvido Perea-García, Vojtěch Fiala, Marta Sibierska, Anna Szala, Dariusz P. Danel

**Affiliations:** 1 Department of Human Biology and Cosmetology, Wroclaw University of Health and Sport Sciences, Wrocław, Poland; 2 Center for Language Evolution Studies, Nicolaus Copernicus University in Toruń, Toruń, Poland; 3 University Institute for Health and Biomedical Research, University of Las Palmas de Gran Canaria (ULPGC), Las Palmas, Spain; 4 Department of Anthropology, Hirszfeld Institute of Immunology and Experimental Therapy, Polish Academy of Sciences, Wrocław, Poland

**Keywords:** Human behaviour, Biological anthropology, Evolutionary developmental biology

## Abstract

The Apemen Faces Database is a novel and versatile stimulus set designed for research in behavioral biology, evolutionary psychology, and related fields. The dataset comprises 620 photorealistic, artificially generated facial images of 31 generalized hominin models, available in multiple ocular coloration variants (31 hominins x 20 color variants). Each of the 31 facial portraits is paired with geometric morphometric data and norming information that includes perceptual ratings of six constructs (Threat, Sociability, Trustworthiness, Health, Age, and Masculinity). Further, editable .psd files enable easy generation of a wide spectrum of great ape eye phenotypes. The images were designed to be morphologically diverse, sufficiently humanlike to elicit social attributions, yet clearly non-human. This unique “humanlike but not human” design facilitates the study of face perception beyond the boundaries of extant human variation, offering novel opportunities for investigating cognitive and perceptual mechanisms in both humans and non-human primates.

## Background & Summary

**Fig. 1:**
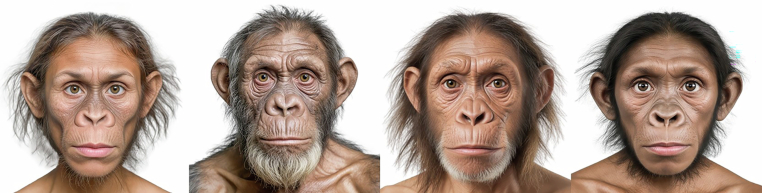
Examples of facial images included in the ApeFD.

**Fig. 2:**
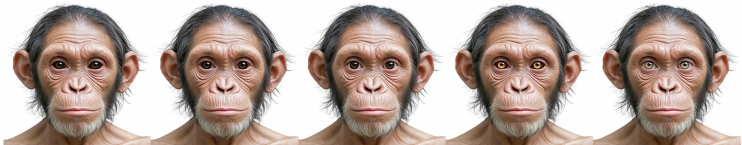
Examples of five ocular morphologies featured in the ApeFD.

We present the ApeFD, Apemen Faces Database^1^, a highly versatile resource with applications in a broad range of research fields, in particular in evolutionary behavioral sciences. Our dataset contains photorealistic, artificial facial images of 31 generalised “hominins” (15 female, 15 male, plus one extra male) presented in different coloration versions (620 PNG files – cf. Figs. 1, 2 for examples), together with geometric morphometric landmarks and measurements, and extensive norming data. It is further extended with 31 PSD master files that allow users to easily arrive at a full range of colorations, capturing most of the extant diversity in visible great ape eye phenotype. The images have been developed to meet the following criteria: (1) represent a diverse range of facial morphologies that (2) look sufficiently humanlike to be meaningfully ascribed human attributes such as sociability or trustworthiness, but (3) be non-human, i.e., distinct from any extant human population. Such “humanlike but not human” stimuli open up possibilities to extend the study of the perception – by humans as well as other primates – of facial features to morphologies outside of the range of human variation.

The intended primary area of application of this dataset is the study of human ocular morphology – that is, studying the range of forms and colorations of the eyes that are perceived as humanlike, as distinct from other great ape species. Features such as very dark sclerae or bright-yellow irises, which are typical of chimpanzee eyes, look highly unnatural in humans; because of that, human faces edited to have dark sclerae or yellow irises cannot be easily used as stimuli, since this instantly attracts the participants’ attention to the manipulation. However, our research^2^ suggests hominid-like stimuli pass as natural when presented with ape-like or human-like coloration. By manipulating scleral and iridal coloration beyond what is present in modern-day humans, we can check how these different morphologies affect a range of outcome variables, measured both through ratings (e.g., perceived trustworthiness), psychophysiology (e.g., heart rate, breathing rate, arousal-mediated skin conductance), or behavior (e.g., reaction times, forced choice). This, in turn, informs key hypotheses on the evolution of the peculiar human ocular coloration, and human cognitive evolution more broadly: in particular, the extremely influential but no longer empirically supported “gaze camouflage” hypothesis^3^ and “cooperative eye hypothesis”^4^.

We emphasise at this point that the utility of the Apemen Faces Database^1^ reaches substantially beyond this principal area of interest. Firstly, it enables the study of other facial features in relation to a range of perceptual and psychological phenomena. We note the particular relevance of our research to evolutionary behavioral sciences, where the perception and evaluation of the face is one of the most – and possibly *the* most – extensively studied topics, and databases such as Chicago FD^5^, Face Research Lab London Set^6^, or Bogazici FD^7^ are a staple resource in a large number of experimental studies. The ApeFD presented here complements these existing databases by making it possible to investigate features, or feature configurations, that fall outside extant variability in our species while still being perceived as humanlike. This aligns with the interest of evolutionary behavioral sciences in identifying human evolved biases in perception, here specifically related to the perception of faces. For example, the database can be used to research features or feature complexes that modern humans associate with masculinity and femininity beyond stereotypes based on standard human facial morphology^8^. Likewise, it can be applied to determine the facial characteristics that predict perception of threat, dominance, and fighting ability^9^.

The need for such a facial database is especially evident considering a number of recent publications. For example, Wacewicz *et al*.^2^ and Wolf *et al*.^10^ both relied on artificial human-like stimuli to circumvent limitations such as violation of expectations. While these stimuli could serve the specific purposes of the studies for which they were designed, their general use is limited. In Wacewicz *et al*.^2^, the stimuli were obtained by morphing together photographs of reconstructions of hominids, which are typically copyrighted. Similarly, Perea-García *et al*.^11^ employed photographs of diverse primate species to alter their appearance beyond the naturally occurring, as primates are not a familiar percept to most human participants. In Wolf *et al*.^10^, the stimuli depicted highly stylized “aliens” that were specifically designed to test children and are unlikely to appear human or believable enough for adult participants. In addition to these factors, the supplementary material, i.e., the norming data and geometric-morphometric measurements, makes the ApeFD a resource for out-of-the-box analyses with a standardized set that will enable repeatable studies.

Finally, the broader applicability of the Apemen Faces Database^1^ extends beyond evolutionary behavioral sciences. In primatology, this stimulus can be feasibly used in studies with non-human primates in zoos^12,13^. In ethnology and cultural anthropology, the database presented here has applications in studying the natural human tendency to alter facial appearance, as it does in the area of cosmetics and plastic surgery, and more broadly, in theatre, video game industry, and film studies^14–17^. In cross-disciplinary research, the database will find applications in researching perceptual phenomena such as gestaltive face perception and the “uncanny valley”^18,19^.

In sum, the Apemen Faces Database^1^ constitutes a high-quality, versatile stimulus set, openly available to the academic community. Although developed primarily as a means of studying aspects of ocular appearance, it can be productively applied to other features or contexts in evolutionary psychology and, even more broadly, in human sciences.

## Methods

### Production of the images

The facial images used in this study were generated by an experienced graphic artist through a combination of manual editing with software tools and automatic image generation with AI-based tools.

### AI-based image generation

Midjourney was employed to create novel, original hominin-like faces, which were then further processed and refined using GetImg.ai. Paid subscription plans were used to make the images eligible for open sharing under the CreativeML Open RAIL-M licence. The final images were produced using the RealVisXL V4.0 model (https://huggingface.co/SG161222/RealVisXL_V4.0) with 45 sampling steps and the Euler sampler, through standard text prompting with iteration, and with iterated admixture of previously generated images as “image reference”. The construction of the prompts revolved around several central elements, in particular the individual (“ancient hominin”, “apeman”, “Australopithecus”), parameters of the photograph (“passport shot”, “biometric passport photograph”, “en-face”, “looking straight”) and quality and realism (“ultra-realistic”, “individual imperfections”). Negative prompts emphasised avoiding artificial and rubbery look, gloss and shine, and artistic effects.

### Prompt examples

- Prompt: *Create a passport-style, full-head portrait of a man, woman, or adolescent resembling an ancient hominin (Australopithecus, Homo habilis, caveman) with smooth matte skin featuring a few shallow wrinkles. The subject should have an intense gaze directed straight at the camera, illuminated by a single symmetrical studio key light to emphasize the raw and primitive essence of prehistoric features. The background should be light grey*.
  Negative prompt: *Avoid rim light, catch light, dark backgrounds, blurriness, plastic or shiny textures, and silicone-like appearances*.
- Prompt: *Create an ultra-realistic biometric passport photograph of a lifelike Australopithecus, against a white background. Capture this ancient hominin with meticulous accuracy, emphasizing finely textured skin with visible pores; nuanced, unevenly placed wrinkles; and random patches of long, dirty, dishevelled hair. Incorporate photorealistic detailing featuring asymmetries to enhance authenticity. Apply individual imperfections, such as blemishes and stains*.
  Negative prompt: *artistic, enhanced, stylized, wax, reconstruction, shiny skin, glossy, special effects, rubber, doll, replica*.
- Prompt: *Design a realistic biometric passport shot of an ape girl, a young female character from the “Planet of the Apes”. Juvenile, feminine face. White background, whole face visible, zoom out. Careful attention to every intricate detail: finely textured skin with noticeable pores, expressive eyes, and subtle, irregular and asymmetric wrinkles. Maintain a level of photographic realism that rivals professional, high fidelity photography of human faces*.

Negative prompt: *truncated, make-up, lipstick, artistic, enhanced, stylized, wax, reconstruction, shiny skin, glossy, special effects, rubber, doll, replica, reflexes, highlights, filters*.

### Image selection

A total of about 7000 images were generated, with a majority rejected as clearly being of insufficient quality, insufficiently realistic, overly apelike, overly humanlike, incorrectly zoomed, or incorrectly positioned. A total of 1245 images were retained to form the initial dataset.

From the initial set of 1245 images, a final set of 31 images, consisting of 15 female and 15 + 1 male representations, was selected through an internal laboratory voting process conducted by four researchers with expertise in biological anthropology, facial morphology, and image analysis (SW, VF, JOPG, DPD). The selected images then underwent a structured post-processing workflow to ensure consistency in visual presentation.

### Standardized post-processing workflow

All images underwent a multi-stage enhancement and editing process by a highly experienced graphic artist (ZL; cf. Fig. 4). Topaz Photo AI v3.5 (topazlabs.com/topaz-photo-ai) was used to enhance facial detail, upscale and sharpen the images, and improve skin texture. Luminar Neo 1.23 (skylum.com/luminar-neo) provided additional skin texturing and mattification when required. Adobe Lightroom Classic v14.1 was employed for global and local adjustments, including symmetrical lighting corrections, softening of deep wrinkles, modifications to contrast, shadow, and black levels, as well as color cast corrections and hair fringing adjustments. Further advanced modifications were made in Adobe Photoshop v26.2, including removal of rim lights and stray light spots, elimination of stray hairs, corrections to hair asymmetry, and adjustments to eye dimensions to enhance a non-human appearance. Background alterations and replacements were also performed in Photoshop, and layered PSD files were created to allow dataset users to flexibly modify the appearance of the iris and sclera (see “Usage notes”).

Final images were saved in PNG format with an sRGB color profile at 300 dpi and a resolution of 1500 × 1500 pixels. Lighting and shadows were standardized across all images, and any remaining color casts or white balance inconsistencies were manually corrected. Skin textures were further mattified to remove excessive shine. Each image was upscaled using Topaz Photo AI v3.5, sharpened, and resized to its original dimensions to ensure consistency in resolution and detail.

**Fig. 3:**
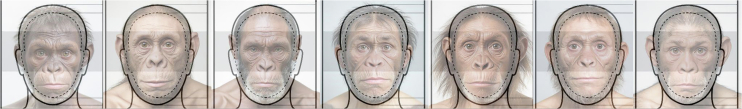
Aligning the position of the faces to a standardised passport outline.

To maintain proportionality, all faces and shoulders were aligned according to a standardized passport outline (see e.g., gov.uk/photos-for-passports/photo-requirements for details), as shown in Fig. 3. A version of the stimulus with the shoulders removed was created, but was rejected as the faces with the shoulders removed were judged as less viable (less natural and less comparable to standard face databases).

**Fig. 4:**
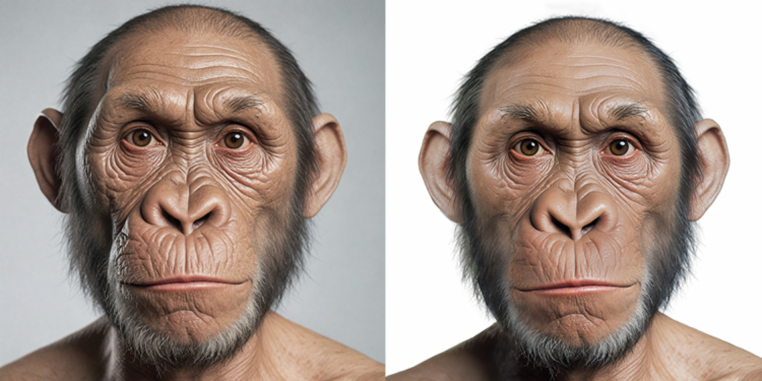
Individual M09 in the initial dataset before post-processing (left) and in the final dataset after post-processing (right). The changes applied manually by a graphic artist involved: aligning and adjusting to fit the passport photo template, removing the original background using advanced cut-out techniques and refining the hair; adding a uniform pure white background; leveling the pupils for symmetry; reducing asymmetrical lighting and shadows; minimizing rim lighting and stray light spots; doing subtle corrections to the shape and illumination of the ears; enhancing overall lighting, contrast, and shadow consistency; doing minor color adjustments; mattifying skin gloss; upscaling and restoring skin texture and fine details, then resizing to the final dimensions; removing blurriness and improving sharpness.

Adjustments were made to the iris brightness to reduce inconsistencies between both eyes in an individual. Lips were retouched to ensure a neutral, closed-mouth expression, and coloration adjustments were applied where needed. On most faces, eye fissure shape was modified to achieve width-to-height proportions intermediate between those typical of humans (relatively wider) and non-human apes (relatively higher). Each face was isolated from its original background, and four different background versions were generated: pure white, mid-grey, black, and black with refined hair edges to eliminate white or grey fringing.

Further refinement was achieved through the addition of adjustment layers for iris and sclera modifications. A “diffuse” sclera version was created with corresponding layers and masks to facilitate targeted modifications – i.e., a patchily pigmented scleral area found in many bonobos and occasionally in other great apes. The final set of images underwent refinement to ensure consistency while preserving realism and precision in facial representation. While complete repeatability of all studio conditions remains unattainable with current AI-based tools, and slight variations may still be present across the final images, the whole dataset preserves a relatively uniform visual style.

### Geometric-morphometrics

Human facial shape is a key to individual identity^20^, but its variance, too, predicts first impressions systematically: studies point to a preference for more “average” and “sex-typical” facial configurations^21^. The dataset is thus provided together with a list of landmark-based facial shape measures, to allow the researchers to account for facial variance other than in the eye area. Each face was labeled with a predefined list of 36 landmarks (denoting anatomically or geometrically identical points across the specimens) and 36 semilandmarks (denoting curves between the landmarks) using an automated tool for placing landmarks on facial portraits, faceDig (www.facedig.org)^22^. Following correction of the automatically placed landmarks, the configurations were subjected to generalised Procrustes analysis via the ‘gpagen’ function in the R package geomorph^23,24^. Subsequently, we calculated the following measures:

1. *Distinctiveness*. It measures how distant a specimen is from the corresponding mean. The lower the number, the more average the apemen are among other apemen. It was calculated separately for male and female stimuli^25^.
2. *Sexual Shape Dimorphism* (SShD) measures the expression of morphological differences between male and female specimens^26^. During calculation, each face is projected on an axis that connects the male and female averages and is assigned a score of SShD, corresponding to its position on the axis. During the calculation, one sex is assigned −1 and the other 1 (default indexes in a linear model).
3. *SexTypicality Score* (SST). For better interpretability, we provide SST, a scaled SShD (zero mean, variance unity), multiplied by −1 in females.
4. *Facial asymmetry*. The landmarks from the left and right parts of the specimen were mirrored along the vertical midline axis, and the paired landmarks from the left and right sides were relabelled. Subsequently, we computed Procrustes distances between the mirrored and the original configurations. Coefficients with higher values indicate less symmetrical faces, i.e., higher facial asymmetry^27^.

**Fig. 5:**
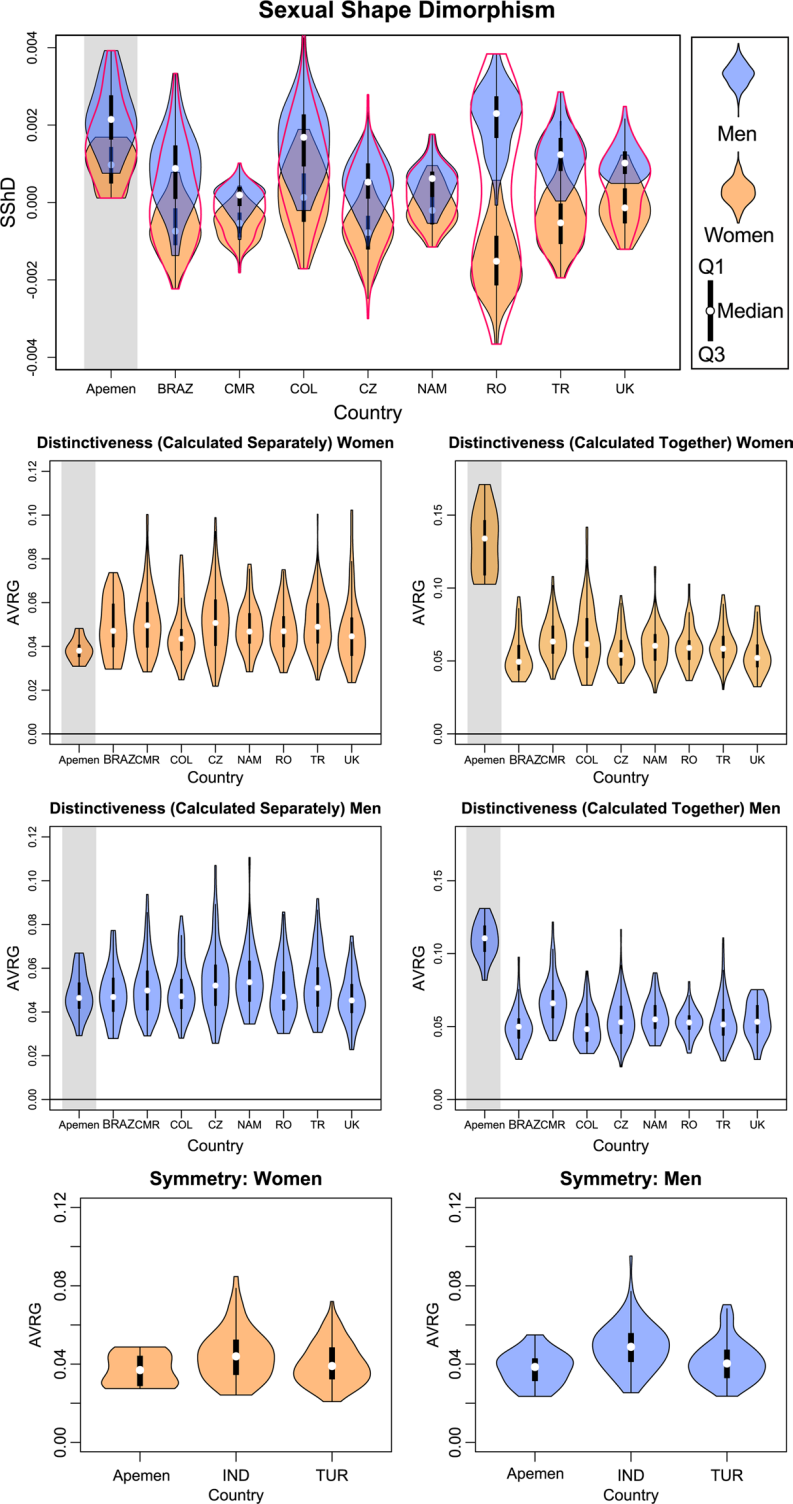
Comparison of morphometric variables in apemen (leftmost column in every plot) and selected other cultures, representatives of the contemporary human population. Symmetrised landmarks for plots in the first to third row were taken from^26^, unsymmetrised coordinates for the last row plots come from^5,7^.

Furthermore, to provide a comparison of the apemen database and contemporary human population, we used the files of facial landmarks from several scientific databases of contemporary human frontal facial photographs^5,7,26^. Figure 5 below compares extant human populations and our database.

### Norming data

We used the Labvanced online application to collect perceptual ratings for each of our 31 facial images on six constructs: Threat, Sociability, Trustworthiness, Health, Age, and Masculinity. Participants were crowdsourced via the online platform Prolific and were remunerated for completing the task (1.8 GBP). Participants were required to have normal or corrected to normal vision and fluency in English, and could use desktop or laptop computers, but not tablets or smartphones. The task was described as studying “how we perceive the faces of prehistoric ancestors of modern humans (so-called hominins, i.e., “apemen” or “cavemen”)”. Informed consent was obtained from all participants. Ethical approval was obtained from the Research Ethics Committee of the Faculty of Philosophy and Social Sciences, Nicolaus Copernicus in Toruń, decision 40/2024.

Mindful of well-described issues with crowdsourcing participants through online platforms like Prolific, we employed a number of mechanisms to ensure the quality of our ratings:

- *eligibility criteria*. We used custom screeners that required potential participants to have completed at least two previous Prolific studies, have a 100% approval rate on previous studies, and have filled in their Prolific profiles with information on a number of standard demographic variables. As a result, the pool of available participants accounted for ca. 29% of all active Prolific users (69,672 out of 242,201);
- *instructions and information on attention checks*. In the study description on Prolific, potential participants were informed that the study contains attention checks, and they were instructed to complete a screen calibration procedure prior to enrollment. The instructions in the study proper began with a screen calibration test and again emphasised the presence of attention checks in the design;
- *sample size and variety*. After piloting the study with 8 participants, we collected a further 160 complete contributions (a further 23 participants “returned”, i.e., withdrew from the study; 4 timed out). Aiming at geographical diversity, we invited participants in 4 batches, at 00:00 CET, 07:00 CET, 12:30 CET, and 18:00 CET. As some participants opened the survey repeatedly, this left us with 184 unique participant IDs;
- *excluding incomplete submissions*. We excluded all the duplicated participants and participants who did not finish the survey completely (33) or rated all the stimuli with the same number (1). This left us with 150 participants.
  Data is provided as “Complete_Individual_Ratings_Before_Excluding_Incongruent_Raters.csv”.
  Of these, 79 identify as men, 68 as women, 2 are not binary, and 1 decided not to say.
  Participants were born in 30 different countries, resided in 22, and possessed nationalities of 27 countries distributed all across the world. Most participants were born/resided in these nine countries: South Africa (29/34), the United States (33/36), the United Kingdom (25/25), Poland (9/9), Portugal (8/8), Canada (2/7), Mexico (3/4), Greece (4/4), and India (4/3).
  The participants were, on average, 32.5 years old (SD = 12.2, range 18–73). The vast majority of participants reported English as their first language (103), followed by Portuguese (9), Spanish (8), Polish (8), and Greek (3).
- *screening for rating consistency within participants (test-retest)*. Participants rated all 31 faces twice: first in round one and then again in round two of the study, so as to monitor their rating consistency. We rejected 15 of the 150 complete contributions with relatively the highest differences between the two rating runs (see “Technical Validation” below).

Data is provided as “DataClean_Individual_Ratings.csv”. Per-face norms based on these 135 participants are provided as “Norms_average_per_face.csv”.

### Rating procedure

**Fig. 6:**
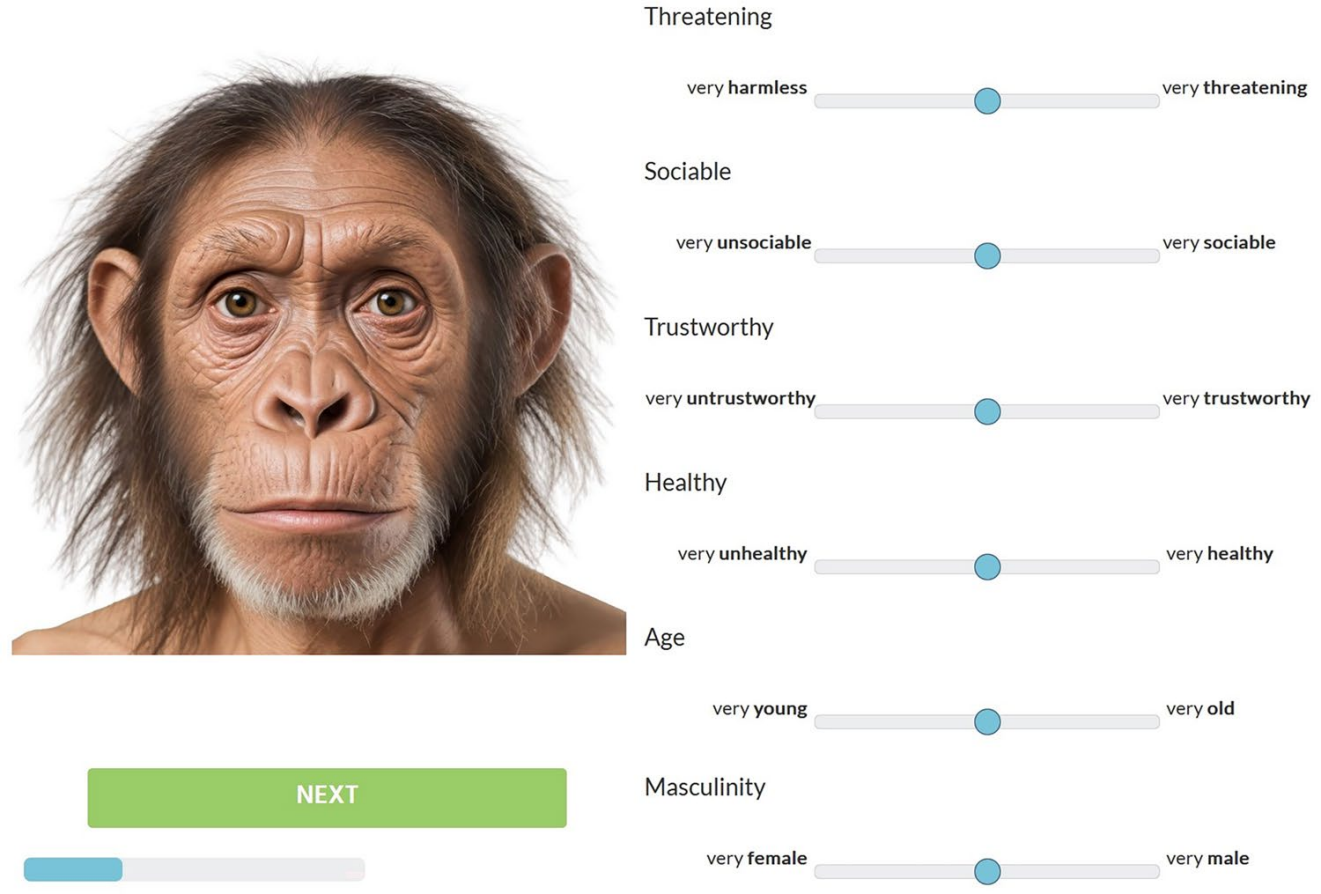
Layout of a rating trial, print-screen from the original survey. The blue horizontal line below the “Next” button represents the progress bar.

Participants rated the faces on six dimensions—(perceived) threat, sociability, trustworthiness, health, age, and masculinity—by moving sliders on a horizontal line between the left (0) and right (100) extremes for each trait. No time limit was set for completing the ratings. The extremes of the scale had verbal labels (e.g., “very young” and “very old” for Age), but the underlying numerical values were not made visible to the participants (cf. Fig. 6). This assessment method drew on the adaptation of the visual analogue scale. It provided a large spectrum of possible responses, which allowed the detection of minute changes in the ratings while assessing multiple attributes and minimized the clustering of ratings around one value, as could be the case with categorical scales.

Participants were also asked to provide free-text answers to three (one after round 1, two after round 2) general questions about the study. Overall, these free-text comments revealed that participants found the study to be interesting and straightforward. Participants reported that their decisions were influenced primarily by facial expressions, eye characteristics, and minor changes in features like skin tone or hairstyle; two participants mentioned a non-facial feature (the shoulders). Some participants reported second-guessing their assessments due to the range of features presented, while others found certain traits, such as sex, particularly difficult to determine. The subjective nature of personality judgments was highlighted, with some mentioning the challenge of maintaining consistency.

### Interrater agreement

We calculated a measure of inter-rater agreement for each scale in Task 1, Task 2, and the combined sample. For the agreement in Tasks 1 and 2 separately, we used Interclass Correlation (ICC;2,k), i.e., two-way random, average score ICC, which measures inter-rater consistency^28^ and is recommended because raters are usually considered representative of a broader population of potential raters. All of our participants saw all of the stimuli. In both tasks, the ICC is higher than 0.96 for every scale.

## Data Records

The dataset has been published in a general-purpose repository for open research data, RepOD^1^ (https://repod.icm.edu.pl/), based on Dataverse, under the permanent identifier: 10.18150/L2RHIA. It consists of the following files:

IMAGES

- 31 .zip files, each comprising 23 files relevant to a given face:
  - 1 .jpg file with a basic version of a face
  - 1 .png file with a basic version of a face
  - 20 .png files with different versions of a face: 5 eye colorations * 4 backgrounds
  - 1 .psd file

GEOMETRIC MORPHOMETRICS

- 2 .tps files (geometric morphometrics measures for male and female faces: ApeFD_Males.tps, ApeFD_Females.tps)
- geometric morphometrics values (ApePPL_GMM_Scales.csv)

NORMING DATA

- Norming data (Norms_Average_Per_Face.tab)
- Raw norming data (RawData_Labvanced.csv) and raw demographic data of the participants (DemographicData_Prolific.tab)
- Processed norming data (DataClean_Individual_Ratings.csv and Complete_Individual_Ratings_Before_Excluding_Incongruent_Raters.csv)

CODE

- R script for merging demographic and norming data (ApeFD_Script.R)

## Technical Validation

Norming data: initially, 150 participants fulfilled the basic criteria (opened the survey just once, rated all the faces twice, submitted the survey correctly following completion, and did not use the same number for all the targets). We computed the average difference between ratings in the first and second run, and marked 10% of participants with the highest average difference. The marked participants (10% with the highest average difference) were subsequently excluded from the dataset. This left us with 135 participants.

## Usage Notes

The database includes 31 layered PSD (Photoshop Document) files that allow users to change the color of the sclera and the iris of the faces, thus tailoring the stimuli to the specific requirements of their studies. Each PSD file includes multiple editable layers, organized and named according to their function. Users can control layer visibility using the eye icon next to each layer in the Layers panel.

### Background selection

Each PSD file contains four interchangeable background layers, of which only one should be enabled at a time:

- White: A pure white background (RGB: 255, 255, 255).
- Grey: A neutral grey background (RGB: 127, 127, 127).
- Black 1: A black background (RGB: 0, 0, 0) with visible hair details, which may include some edge fringing.
- Black 2: A black background (RGB: 0, 0, 0) using a soft mask to reduce fringing around hair edges.

Users are encouraged to select the background most appropriate for their experimental design and ensure consistency across stimuli within a given condition.

### Iris adjustment layers

Several layers are provided to allow for systematic manipulation of iris appearance:

- Dark iris multiply: Applies a darkening effect to the iris using the *Multiply* blending mode. The effect can be adjusted using the layer’s opacity setting (0–100%). To refine the area of application, edit the associated layer mask using a black or white brush.
- Bright iris screen: Brightens the iris using the *Screen* blending mode, similarly controlled via opacity settings. As with other layers, the mask can be manually adjusted for precision.
- Hue/saturation orange iris: An adjustment layer modifying the color properties of the iris. Users may adjust the *Hue*, *Saturation*, and *Lightness* sliders to achieve the desired color characteristics. This layer is designed to be used in conjunction with either the Bright or Dark iris layers. The layer’s overall effect can also be modulated using opacity and mask refinements.

### Sclera adjustment layers

The sclera can also be manipulated through several dedicated layers:

- Sclera multiply: Darkens the sclera using the *Multiply* blending mode. The effect can be customized via opacity and mask editing.
- Diffused sclera: Includes two separate layers corresponding to the left and right eyes. These layers use soft masks to allow for diffusion of the scleral area. Their shape and position can be manually adjusted or transformed. Additional refinement can be achieved through mask painting.
- Sclera subtract: Applies a pronounced darkening effect to the sclera using the *Subtract* blending mode. Due to the intensity of this mode, it is recommended to reduce opacity to achieve more natural results. Edges can be softened by editing the mask range.
- Hue/saturation sclera: An adjustment layer for modulating scleral color. This can be used with the above scleral layers to fine-tune *Hue*, *Saturation*, and *Lightness*. For optimal results, this layer should be blended with other scleral layers using varying opacity levels.

### General recommendations

Researchers are encouraged to experiment with different combinations of blending modes, opacity levels, and masks to achieve the desired visual outcome. In many cases, adjusting individual layer masks using a soft brush with customized opacity and flow settings may yield a more realistic appearance, especially for subtle modifications of the eye region. This level of control is intended to accommodate diverse research contexts, such as manipulating facial expressions, gaze perception, or morphological realism. For best practices, users should document any modifications performed on the base stimuli and ensure consistency across all manipulated images within a given study.

## Data Availability

All data and code for this study are available in RepOD^1^, under the link repod.icm.edu.pl/dataset.xhtml?persistentId = 10.18150/L2RHIA.
